# Measuring quality of life of primary antibody deficiency patients using a disease-specific health-related quality of life questionnaire for common variable immunodeficiency (CVID_QoL)

**DOI:** 10.1186/s41687-019-0101-x

**Published:** 2019-02-26

**Authors:** Jintana B. Andersen, Knut Midttun, Kristin J. B. Feragen

**Affiliations:** Centre for Rare Disorders, Oslo University Hospital, Rikshospitalet, Postboks 4950 Nydalen, 0424 Oslo, Norway

**Keywords:** Quality of life, Primary antibody deficiency, Survey and questionnaire, Common variable immunodeficiency, Psychometrics, Patient-reported outcomes, Reliability, Validity, Disease-specific

## Abstract

**Background:**

Common variable immunodeficiency (CVID) and other primary antibody deficiencies (PAD) are a heterogeneous group of > 300 congenital disorders affecting the immune system. Until recently, efforts to measure health-related quality of life (QoL) in PAD patients have utilised generic QoL tools and disease-specific tools for other conditions. Still, the full impact of the disease is probably not understood. We evaluated the performance of the CVID_QoL, a novel disease-specific QoL instrument for adults with CVID, on Norwegian PAD patients and compared the results to those of the generic WHOQOL-BREF.

**Methods:**

Respondents were recruited through the Norwegian Centre for Rare Disorders’ patient database. Included patients fulfilled the following criteria (all three): 1.) Age ≥18 years, 2.) a PAD diagnosis, 3.) currently on immunoglobulin therapy. The CVID_QoL is a 32-item questionnaire. Global CVID_QoL scores were compared between Norwegian PAD patients and Italian CVID patients.

**Results:**

In total, 83 PAD patients filled out the CVID_QoL, 63% had CVID, 76% were females. 32 patients filled out the WHOQOL-BREF. Feasibility was high (<1% missing). Internal consistency for the emotional- (Cronbach’s α-value = 0.91) and relational functioning (α =  0.77) subscales was high, but questionable for the gastrointestinal and skin symptoms subscale (α =  0.66). Convergent validity varied from weak to strong (range 0.3–0.8). Floor and ceiling effects were present.

**Conclusions:**

Although many disease-specific characteristics are probably shared with CVID and other PAD, the CVID_QoL captures some, but not all, dimensions of PAD patients’ QoL. More evaluations of the CVID_QoL’s performance in different contexts are needed.

**Electronic supplementary material:**

The online version of this article (10.1186/s41687-019-0101-x) contains supplementary material, which is available to authorized users.

## Introduction

Primary antibody deficiencies (PAD) are the most frequent primary immunodeficiency, where common variable immunodeficiency (CVID) is the most common clinically relevant diagnosis, characterized by severe and chronic infections [1]. Although immunoglobulin (Ig) replacement therapy has greatly improved the morbidity and mortality of PAD patients over the past decades [2, 3], it seems less effective on other PAD-associated challenges such as autoimmunity, malignancy, recurring gastrointestinal disorders, and chronic lung complications [4]. The true incidence of PAD is unknown [3], and primary immunodeficiency disorders as a group might not be as rare as previously thought [5].

The psychological and social impact of having a rare diagnosis may differ from having a more common medical disease, due to a lack of knowledge both in society and in health care [6, 7]. The attention to the patient experience of health-related quality of life (HRQoL) as a factor influencing the individual’s well-being and treatment choices are increasingly explored by clinicians and researchers [8–11]. HRQoL is a concept that encompasses physical, psychological and social aspects of well-being, and may be assessed through patient reported outcome measures (PROMs) [12]. Systematic QoL assessments have become important guides to improved targeted clinical care and patient outcomes. Generic QoL instruments have the advantages of assessing a broad range of populations and conditions [10]. However, the true impact of specific conditions may be lost. Disease-specific tools may help evaluating the QoL for patients with a particular disease more precisely than generic tools alone [13–15].

Validated disease-specific tools exist for many different conditions. A condition specific measure was lacking for patients with PAD until recently [16], and previous efforts to measure the QoL of these patients have been through generic questionnaires and/or disease-specific tools developed for other conditions [17–25]. Given the clinical spectrum of PAD, specific tools for other diseases may be applicable to some extent, such as the Asthma QoL Questionnaire [26]. Nevertheless, the impact of patients with PAD’s QoL is probably not fully understood. In 2016, the CVID_QoL questionnaire, the first condition-specific instrument for measuring QoL in adult patients with CVID was developed and initially validated by Quinti and colleagues [8]. According to the authors, the CVID_QoL was able to identify relevant characteristics to patients with CVID. However, there is a need for further evaluations of the performance and validity of CVID_QoL across different populations of patients with PAD.

The validity of QoL instruments is determined by the quality of its measurement properties. Several quality criteria for the evaluation of QoL questionnaires are available [27]. The consensus-based standards for the selection of health status measurement instruments (COSMIN) checklist is a useful tool in evaluating the methodological quality of studies on measurement properties [28, 29]. To our knowledge, the psychometric properties of CVID_QoL have not yet been widely tested in different contexts, populations and disease characteristics. In this study, we aimed to critically appraise the CVID_QoL questionnaire in a cohort of Norwegian adults with CVID and other PAD using the COSMIN checklist as a guideline, and comparing the CVID_QoL questionnaire’s performance with the generic World Health Organization’s Quality of Life Scale Brief version (WHOQOL-BREF) questionnaire [11].

## Methods

### Sample

**Fig. 1:**
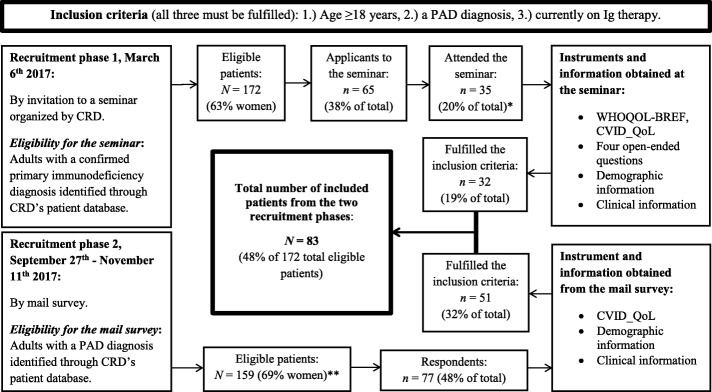
Flowchart of the recruitment process and survey administration. Abbreviations:* PAD* = primary antibody deficiency, *Ig* = immunoglobulin, *CRD* = Centre for Rare Disorders. *One patient did not consent to the study.**This group overlapped with the 172 eligible patients from the recruitment phase 1

The Centre for Rare Disorders (CRD) is an interdisciplinary, nationwide competence center in Norway (5.3 million inhabitants). The CRD provides information, counselling, seminars and research, covering more than 70 rare disorders, including CVID and other PAD. Most patients attending our seminars or receiving information, counselling or follow-up of any kind from the CRD are registered in our patient database with contact information and diagnosis. The registration in our patient database is based on oral consent. All study subjects were identified through our patient database. To be included in the study, all three of the following inclusion criteria had to be fulfilled: 1.) Age 18 years or older, 2.) registered with a PAD diagnosis in our patient database, and 3.) currently on Ig replacement therapy. The study subjects were recruited on two separate occasions (Fig. 1).

The first sample was recruited at a CRD seminar held in March 2017 for adult patients with a PAD diagnosis. Eligibility for the seminar was an assumed ability to benefit from the seminar, and personal history of CRD seminar participation (first-timers prioritized). All participants recruited through the seminar were asked to fill out both the CVID_QoL and WHOQOL-BREF questionnaires. Four open-ended questions were included in the package, including the study subject’s comments on the questionnaires. Demographic information obtained included age, educational level, marital and occupational status and responsibility for children. Patient reported clinical information included main type of treatment, including administration route of Ig replacement therapy.

The second sample was recruited in the period of September to November 2017 by mail. Eligible individuals were all patients 18 years or older who had a registered PAD diagnosis in our patient database. These patients were asked to fill out the CVID_QoL questionnaire only. They were also asked to include information about their weight, height and time of diagnosis, but administration route of Ig replacement therapy was not included. The recruitment process and survey administration are shown in Fig. 1. For patients who filled out the CVID_QoL both at the seminar and in the later mail survey, the data from the first context were used, unless specified otherwise.

### Measures

The CVID_QoL is a self-reported, disease-specific questionnaire, containing 32 items which are calculated globally and across three dimensions [8]: Emotional functioning (EF) (13 items), relational functioning (RF) (9 items) and gastrointestinal and skin symptoms (GSS) (4 items). Each item is rated on a 5-point scale (0 = “never”, 4 = “always”). There are no negatively worded items, and higher values generally indicate higher degree of disability. The questionnaire takes about 10–15 min to complete. The sums of the global and dimensional scores are transformed to a percentage of the maximum possible score. Scores of missing items are imputed as average scores for the same dimension when less than three answers are missing. The questionnaire was originally developed in Italian, and then translated to English through a standard procedure [8]. Permission to use the CVID_QoL was given to CRD by the Italian authors. The English version of the CVID_QoL was adapted to Norwegian in a process that included translation and back-translation using in-house resources (Additional file 1: Table S1). However, a formal validation of the Norwegian version was not performed.

The WHOQOL-BREF is a self-reported, generic abbreviated version of the WHOQOL-100 questionnaire [11] and contains 26-items. The first two items address self-perceived quality of life (item 1) and health satisfaction (item 2). These are not included in the domain scores. The remaining 24 items are grouped into four domains pertaining to QoL: Physical health (7 items), psychological (6 items), social relationships (3 items), and environment (8 items). Each item is rated on a 5-point scale (from 1 to 5). Higher scores indicate higher QoL. The questionnaire takes about 10–20 min to complete. Mean scores are calculated for each of the domains. Domain scores are not calculated when a specified number of answers are missing, and if > 20% of the data is missing, the entire case is discarded [30]. The WHOQOL-BREF has been validated for Norway, and general population data are available [31].

### Analyses

Reliability is the degree to which scores are free from random error, in which interrelatedness among the items reflects the internal consistency [28]. Internal consistency was assessed by calculating Cronbach’s alpha values for the three CVID_QoL dimensions. The minimal accepted standards for alpha coefficients for group comparisons are 0.7 [27]. Convergent validity reflects the extent to which two measures capture a common construct [32] and was examined by using Pearson’s correlation analysis for CVID_QoL and WHOQOL-BREF scores. Proxies of item relevance and comprehensiveness were assessed by calculating missing item responses, floor and ceiling effects and qualitative interpretation of open-ended questions [28]. Floor and ceiling effects were defined as present if the lowest or highest possible score were achieved by >15% of the study subjects [27].

Selected CVID_QoL measurement properties were analyzed and scores calculated, comparing our results with those reported by Quinti et al. [8]. Correlations between CVID_QoL scores and WHOQOL-BREF scores were calculated. The distribution was tested for normality by Shapiro-Wilk’s test, calculating *Z*-values for skewness and kurtosis, and visual assessments of histograms, box- and Q-Q plots. Independent samples *t*-test and Oneway analysis of variance (ANOVA) were used to compare means between groups as appropriate. *P*-values < 0.05 were considered statistically significant. For comparisons of means where external data, i.e. the Norwegian reference population [31], were used, an online calculator for independent samples *t*-test from GraphPad QuickCalcs was utilized (https://www.graphpad.com/quickcalcs [accessed May 2018]). All other statistical analyses were performed in SPSS version 23.0.

## Results

In all, 83 patients (32 from the seminar, 51 from the mail survey) with PAD participated (57% of the total number of registered patients with PAD in the CRD’s patient database). Of the participants, a majority was females (76%), had a CVID diagnosis (63%), and had a body mass index (BMI) within the normal range (47%). Mean age was 47 years (SD 15.9, range 18–83 years). Over half (54%) had more than 13 years of education and about a quarter (25%) had children under 18 years. Mean duration of disease of the patients from the mail survey was 15.2 years (SD 12.1, range 0–48 years). Of those asked to report administration route of Ig therapy (*n* = 32), 88% were on self-administered subcutaneous Ig. There were no differences between the two sub-samples of the Norwegian cohort with regard to gender, age and level of education (Additional file 2: Table S2). Demographic and clinical information with comparisons to the Italian study sample [8] are presented in Table 1.

**Table 1:** Demographic and clinical characteristics of the Norwegian and Italian [8] cohorts

	Norway *N* (%)	Italy [8] *N* (%)
Demographic characteristics		
Total	83 (100)	118 (100)
Male	20 (24)	46 (39)
Female	63 (76)	72 (61)
Mean age (years, *SD*)	47 (16)	*NA*
Age ≤ 50 years	45 (54)	66 (56)
Age > 50 years	38 (46)	52 (44)
Education ≤13 years	37 (45)	31 (26)
Education > 13 years	45 (54)	87 (74)
No children < 18 years	61 (74)	*NA*
Children < 18 years	21 (25)	*NA*
Clinical characteristics		
CVID	52 (63)	118 (100)
Other PAD	31 (37)	0 (0)
Mean disease duration (years, *SD*)	15 (12)*	12 (11)
SCIG	28 (88)**	13 (11)
IVIG	4 (13)**	105 (89)
BMI ≤ 18,5	3 (4)	9 (7)
BMI 18,6 - 24,9	39 (47)	67 (57)
BMI ≥ 25	27 (33)	42 (36)

*NA* = not available, *CVID* = common variable immunodeficiency, *PAD* = primary antibody deficiency, *SCIG* = subcutaneous immunoglobulin, IVIG = intravenous immunoglobulin, *BMI* = body mass index. *N* is reported as frequencies. Percentages (%) are calculated from the total number of patients in the respective samples unless specified otherwise. Some numbers were rounded so not all percentages total 100%.*Data only available for the patients recruited through the mail survey (*n* = 51).**Data only available for the patients recruited through the CRD seminar (*n* = 32)

### Reliability

High internal consistency was found for the EF and RF subscales with Cronbach’s alpha values of 0.91 (0.82) and 0.77 (0.84), respectively. Values from Quinti et al. [8] are reported in parentheses for comparison. For the GSS subscale, the Cronbach’s alpha value was 0.66 (0.74). An alpha score of 0.77 was achieved when items number 2 (“dietary changes”) and 26 (“skin diseases”) were removed from the GSS subscale. When adding these two items to the EF and RF subscales, the alpha values of 0.91 and 0.81, respectively, were achieved.

### Convergent validity

Consistently negative correlations between the global and dimensional CVID_QoL and the two overall items and four WHOQOL-BREF domains were found, with absolute values ranging from 0.32 to 0.80. The negative values reflect opposed scaling where lower scores indicate better QoL in the CVID_QoL, whereas higher scores in WHOQOL-BREF indicate better QoL. Strongest correlation was found between the WHOQOL-BREF’s physical health domain and the global CVID_QoL scores (− 0.80). Also, high correlation between the WHOQOL-BREF environment domain and CVID_QoL global (− 0.69) and EF subscale (− 0.72) was observed. Table 2 demonstrates the correlations between the two instruments.

**Table 2:** Correlations of the global CVID_QoL scores with scores of WHOQOL-BREF

WHOQOL-BREF (*n* = 32)^a^	CVID_QoL Global	EF	RF	GSS
Item 1 (Patient’s self-rated QoL)	−.61**	−.61**	−.51*	−.55*
Item 2 (Patient’s self-rated satisfaction with health)	−.44	−.44	−.42	−.27
Physical health domain	−.79**	−.80**	−.69**	−.60*
Psychological domain	−.56*	−.58*	−.44	−.48
Social relationships domain	−.50	−.53*	−.32	−.57*
Environmental domain	−.69**	−.72**	−.53*	−.58*

^a^Only available for the patients recruited through the CRD seminar. *EF* = emotional functioning, *RF* = relational functioning, *GSS* = gastrointestinal and skin symptoms.*Correlation (Pearson) is significant at the 0.05 level.**Correlation (Pearson) is significant at the 0.01 level

### Item relevance

Item number 15 (“loss of autonomy”) [8] appeared to be problematic for participants, based on the results for missing items. Evaluations of the open-ended questions indicated that item number 32 (“tired”) [8] was interpreted variably among the respondents, while item number 31 (“troubled by other patients”) [8] was often missing and, from the open-ended questions, viewed as irrelevant by some respondents. During the translation process, the items numbers 7 (“unable to provide care”) and 26 (“skin diseases”) [8] posed particular challenges, although these items had no missing replies.

### Floor and ceiling effects

Overall, about 20% of the replies had the lowest score of “0 = never” and 7% at the highest score of “4 = always”. None of the respondents achieved the maximum score which corresponds to lowest QoL in any of the three dimensions nor globally. One respondent achieved the minimum score, corresponding to the highest QoL, both globally and for the EF and RF dimensions. Seven respondents (8%) also achieved the highest QoL for the GSS dimension. In 20 of the 32 items, > 15% of the respondents used the lowest value “0 = never”, whereas the highest value “4 = always” was used by > 15% of the respondents in 5 of the items.

### CVID_QoL scores

Compared to the Italian cohort [8], the Norwegian patients reported poorer global CVID_QoL scores in total (M = 37.4, SD = 15.3, *p <* 0.001), for males (M = 33.8, SD = 14.4, *p <* 0.05), females (M = 38.6, SD = 15.6, *p <* 0.05), younger patients (M = 37.7, SD = 17.9, *p <* 0.001), patients with higher education (M = 37.2, SD = 12.1, *p <* 0.001), and patients with BMI within the normal range (M = 37.0, SD = 16.2, *p <* 0.05). Comparisons of global CVID_QoL scores between the two samples are reported in Table 3.

**Table 3:** Comparisons of the global CVID_QoL scores for the Norwegian and the Italian [8] cohort

Characteristics	Global CVID_QoL scores, mean (*SD)*		
	Norway *N* = 83	Italy [8] *N* = 118	*p -* value*
Total	37.4 (15.3)	29 (16.5)	< 0.001
Male	33.8 (14.4)	25.7 (14.2)	< 0.05
Female	38.6 (15.6)	31.3 (16.4)	< 0.05
Age ≤ 50 years	37.7 (17.9)	26.5 (15.5)	< 0.001
Age > 50 years	37.1 (11.8)	32.6 (15.7)†	*ns*
Education ≤13 years	37.6 (18.8)	32.1 (17.5)	*ns*
Education > 13 years	37.2 (12.1)	28.3 (15.3)	< 0.001
No children < 18 years	36.4 (14.7)	*NA*	
Children < 18 years	41.3 (16.8)	*NA*	
CVID	36.6 (15.3)	29 (16.5)	< 0.05
Other PAD	38.8 (15.5)	*NA*	
SCIG**	41.1 (15.7)	*NA*	
IVIG**	34.5 (13.5)	*NA*	
BMI ≤ 18.5	39.3 (3.0)	41.1 (11.4)‡	*ns*
BMI 18.6–24.9	37.0 (16.2)	28.2 (15.8)	< 0.05
BMI ≥ 25	35.5 (15.2)	28.0 (15.9)	*ns*

CVID_QoL scores are presented as mean (*SD*). *NA* = not available, *ns* = not significant, *CVID* = common variable immunodeficiency, *PAD* = primary antibody deficiency, SCIG = subcutaneous immunoglobulin, IVIG = intravenous immunoglobulin, BMI=Body mass index.*Comparison of Global CVID_QoL scores between the Norwegian and the Italian cohorts.**Data only available for the patients recruited through the CRD seminar (*n =* 32).†Reported as significantly different from age ≤ 50 years (*p* = 0.04) in Quinti et al. [8].‡Reported as significantly different from BMI > 18.5 (*p* = 0.02) in Quinti et al. [8]. Otherwise, no significant differences were found for the global CVID_QoL scores within the Italian or Norwegian cohorts

When comparing global CVID_QoL scores between subgroups in the two cohorts respectively (Table 3), no significant differences in the Norwegian sample were observed, including administration route of Ig treatment. Compared to the Norwegian population [31], significantly poorer QoL in the WHOQOL-BREF physical health domain was observed for patients with PAD (M = 15.8, SD = 2.8 and M = 13.0, SD = 3.6, respectively, *p* < 0.001).

Missing response rate for all CVID_QoL items was 0.9%. Items number 15 (“loss of autonomy”), 23 (“difficulty in sexual relations”), and 31 (“troubled by other patients”) [8] were most often missing (*n* = 5, 3 and 3, respectively). None of the respondents had > 26 items (80%) missing. In one case, the EF dimension was not computed due to several missing items (< 16/19 questions answered).

## Discussion

The present study evaluated the performance of the CVID_QoL questionnaire in a cohort of Norwegian patients with PAD. We found that the internal consistency was excellent or acceptable for the subscales of EF and RF respectively, but was questionable for the GSS subscale. The GSS subscale measures two of three clinically distinct CVID features, gastrointestinal symptoms and skin disease. However, the subscale consists only of 4 items, three of which are related to gastrointestinal symptoms and one to skin disease. The low number of items combined with the assumption of interrelatedness of skin disease with dietary changes and diarrhea, probably contributes to the poor degree of interrelatedness of the items in the GSS subscale in our cohort. In contrast to the Italian study [8], our cohort consisted of both CVID patients and patients with other PAD, both of which are heterogeneous disorders with wide spectrums of dysfunctional antibody production and clinical characteristics [2, 33]. Diarrhea, both chronic (up to 60%) and infectious (up to 32%), are common features of PAD in general and CVID in particular [34]. Non-infectious diarrhea is associated with increased morbidity and mortality as treatment is often ineffective [35]. Dermatologic manifestations are not as common as diarrhea, and have widely different presentations from very mild to more severe forms [2, 34, 36]. Gastrointestinal symptoms and skin symptoms may thus not belong to the same construct. This might be demonstrated, although two-item scales are considered problematic [37], by removing the items “dietary changes” and “skin diseases” from the GSS subscale, in which an acceptable alpha score was then achieved. Moreover, the alpha values improved from acceptable to good by adding these two items to the other two respective subscales, and were thereby comparable to the values in the Italian CVID population [8].

Another novel disease-specific tool for measuring QoL in PAD patients, PADQOL-16, was developed and published shortly after the CVID_QoL [16]. The study population included patients with both CVID and other PAD. The 16 items of the PADQOL questionnaire included one question related to gastrointestinal symptom (“nausea and bloating”), but none related to skin symptoms [16], suggesting that skin symptoms might not have as great impact of QoL for PAD patients as gastrointestinal symptoms [19], although more evidence is needed.

As expected, CVID_QoL scores correlated strongly with the WHOQOL-BREF’s physical health domain. Surprisingly, this was also observed in the environment domain of the WHOQOL-BREF and CVID_QoL global and EF subscale. Quinti et al. showed good convergent validity for the EF and RF subscales correlating with conceptually similar dimensions of particularly the generic Short Form-36 questionnaire (SF-36) [8]. Although not directly comparable due to differences in populations studied, weak convergent validity between related fields in the WHOQOL-BREF and SF-36 questionnaires have been demonstrated in other studies [38–40]. These findings might indicate that, as SF-36, the CVID_QoL measures aspects related to patient’s health and their functional performance more accurately than WHOQOL-BREF [38]. This variation might however be influenced by the small sample completing the WHOQOL-BREF (one-third of our total cohort).

The content validity of this study was limited by the lack of a validation of the Norwegian version. Several problematic items were identified in the Norwegian version, e.g. the item worded as “loss of autonomy” (number 15) in the English version [8], but was worded as “less independent” in the Norwegian translation, which is a double negation-type statement. Evaluations of the open-ended questions indicated that “tired” (item number 32) [8] was interpreted differently among the respondents, in the sense of “sleepy” by some and as “fatigued” by others. Finally, the wording of item number 26 (“skin disease”) [8] seemed to indicate that everyone responding had a skin problem. Nevertheless, feasibility was high with low missing response rate. Overall, the Italian patients achieved better CVID_QoL scores than the Norwegian patients. Floor and ceiling effects were observed with a tendency towards better QoL. These effects were not examined in the initial validation by Quinti et al. [8], and could indicate that the five-point scale of CVID_QoL might not allow a good enough discrimination between the patients [27], particularly at the low end of the scale. Missing item responses and floor and ceiling effects are merely proxies of item relevence and scale comprehensiveness, thus the content validity of the CVID_QoL needs to be further evaluated.

Several factors might explain the observed differences between the two studies. First, the study subjects in the Norwegian sample are presumably more prone to selection bias compared to the Italian sample. The first group of study subjects was recruited primarily among participants in seminar for patients with PAD. Why some people choose to join a seminar and some do not, is a subject of speculation. Arguably, both the healthiest and the sickest patients would abstain from participating. However, there is some evidence indicating that people with low socioeconomic status and higher prevalence of lifestyle-related diseases are underrepresented in surveys and that non-participants are characteristically less healthy than survey participants [41–44]. The patient demographics in our study lend some support to this as the majority had a higher education and a BMI within the normal range. On the other hand, the proportion of patients with higher education was even higher in the Italian sample than the Norwegian sample (74% vs. 55%, respectively), while the proportions of patients with BMI within the normal range are slightly higher in the Italian sample compared to the Norwegian sample (57% vs. 47%, respectively). Second, the Norwegian sample consisted of both CVID patients *and* patients with other PAD diagnoses with possibly other health-related issues having an impact on their QoL that are not addressed in the CVID_QoL [19, 21, 24, 45]. Third, the proportion of female respondents in the Norwegian sample was very high (76%). Females tend to report worse outcomes in QoL-studies compared to males in different clinical settings and populations [46–48]. Lastly, differences in the distribution of the CVID_QoL global scores between the two samples might further bias the findings. While our scores were normally distributed in our estimation, the distribution in the Italian study seems somewhat skewed as illustrated in Figure 3 in Quinti et al. [8].

This study has several limitations. First, a convenience sampling was used, making direct comparison to the original Italian cohort [8] challenging. Also, clinical information in our study was limited. Important information regarding disease severity, comorbidity, number of acute infections and hospitalizations is thus missing. Such factors have in previous studies been shown to influence the perceived health status [8, 49]. Due to two different recruitment processes, a uniform approach to obtain clinical, demographic and QoL information was not achieved. Structural validity could not be assessed due to the limited sample size. A formal validation of the Norwegian version of the CVID_QoL is needed to enhance interpretability and content validity.

## Conclusions

Patients with PAD are a heterogeneous group of patients with differences in clinical spectrum and severity. Although many disease-specific characteristics are probably shared by CVID and other PAD, the impact on QoL on PAD is probably not fully understood by using CVID_QoL alone. Our findings highlight the need for further evaluations of the CVID_QoL with special attention to the factor structure. Finally, it would be of great interest to compare the performance of the PADQOL-16 and the CVID_QoL to better understand the impact of having PAD on HRQoL.

## Additional files

Additional file 1: **Table S1.** The Norwegian version of the CVID_QoL questionnaire. (DOCX 19 kb)

Additional file 2: **Table S2.** Comparison of demographic characteristics of the two Norwegian sub-samples. (DOCX 15 kb)

## Abbreviations

COSMIN: The consensus-based standards for the selection of health status measurement instruments
CRD: Centre for Rare Disorders
CVID: Common variable immunodeficiency
EF: Emotional functioning
GSS: Gastrointestinal and skin symptoms
HRQoL: Health-related quality of life
Ig: Immunoglobulin
PAD: Primary antibody deficiencies
RF: Relational functioning
WHOQOL-BREF: World Health Organization’s Quality of Life Scale Brief version
