# Supplementary material for: Measuring quality of life of primary antibody deficiency patients using a disease-specific health-related quality of life questionnaire for common variable immunodeficiency (CVID_QoL)
Source: J Patient Rep Outcomes. 2019 Feb 26;3:15. doi: 10.1186/s41687-019-0101-x (PMC6391500; doi:10.1186/s41687-019-0101-x)
Supplement: Supplementary file 2 — Table S2. Comparison of demographic characteristics of the two Norwegian sub-samples. (DOCX 15 kb) [file 41687_2019_101_MOESM2_ESM.docx]

|  | **CRD n = 32 (%)** | | **Mail survey n = 51 (%)** | | ***p-value*** |  |  | |  | |  | |  |
| --- | --- | --- | --- | --- | --- | --- | --- | --- | --- | --- | --- | --- | --- |
| **Gender** |  | |  | | 0.5 |  |  | |  | |  | |  |
| Female | 23 (72) | | 40 (78) | |  |  |  | |  | |  | |  |
| Male | 9 (28) | | 11 (22) | |  |  |  | |  | |  | |  |
| **Age group** |  | |  | | 0.5 |  |  | |  | |  | |  |
| ≤ 50 years | 16 (50) | | 29 (57) | |  |  |  | |  | |  | |  |
| > 50 years | 16 (50) | | 22 (43) | |  |  |  | |  | |  | |  |
| **Level of education** | |  | |  | 0.8 |  | |  | |  | |  | |
| ≤ 13 years | 14 (44) | | 23 (45) | |  |  |  | |  | |  | |  |
| > 13 years | 18 (56) | | 27 (53) | |  |  |  | |  | |  | |  |
| CRD=Centre for Rare Disorders, i.e. course participants. | | | | | |  |  | |  | |  | |  |
| The two sub-samples were similar demographically assessed by chi-square test for categorical variables (*p* > .05). | | | | | | |  |  |  |  |  |  |  |

**Supplementary Table 2.** Comparison of demographic characteristics of the two Norwegian sub-samples.
